# Firearms Injury Prevention, Emergency Medicine, and the Public’s Health: A Call for Unity of Purpose

**DOI:** 10.5811/westjem.2021.4.52861

**Published:** 2021-05-07

**Authors:** Chadd K. Kraus, Mark I. Langdorf

**Affiliations:** * Geisinger, Department of Emergency Medicine, Danville, Pennsylvania; † University of California, Irvine, Department of Emergency Medicine, Irvine, California

We enthusiastically present the *Western Journal of Emergency Medicine* (*WestJEM*) Special Issue on Firearms Injury Prevention. This project is the culmination of several years of discussions, deliberations, and evaluations of peer-reviewed manuscripts.

Critics might call an issue of *WestJEM* focused on firearms-related injury and death as politically motivated or skewed. This issue of *West*JEM is not intended to litigate gun laws or regulations. It is not meant to further divide strongly held views on the topic with blanket proposals for or against legislative or regulatory approaches. While necessary, the spirited discussions of legislative and regulatory measures are beyond the scope of this special issue. On the contrary, we offer a collection of peer-reviewed research, editorials, and perspectives to engage emergency physicians in productive discussions toward practical solutions to reduce firearms-related morbidity and mortality. Papers in this issue provide regional and national perspectives on firearms-related injuries, thought-provoking perspectives on firearms, descriptions of injury patterns and characteristics, and injury prevention and risk reduction strategies such as safe storage. As the editors of this special issue, we hope these papers will move the discussion forward with evidence and expert consensus.

We appreciate that violence and injuries with firearms are one of many public health challenges for emergency physicians, and all of these (e.g., motor vehicle safety, interpersonal violence) merit scientific inquiry, evaluation, and discussion. The response that “knives injure and kill, cars injure and kill, etc…” oversimplifies the morbidity and mortality from firearms and disregards the demonstrated effectiveness of injury prevention research in public health and emergency medicine. Firearms-related research has been a controversial, “hot potato” in the interplay of science and politics, particularly following the 1996 Dickey Amendment that effectively halted federally funded research on firearms if it involved gun control.^1^–^3^ The more controversial the topic, the more we need to engage our objective, scientific inquiry, and the less we should rely on emotion. We hope that this issue will be thought-provoking and productive.

As in broader society, the mere mention of firearms is potentially divisive among emergency physicians, with approximately 40% of members of the American College of Emergency Physicians (ACEP) owning firearms.^4^ The American Board of Emergency Medicine 2019 Model of the Clinical Practice of Emergency Medicine recognizes “firearm injury prevention” among the evolving trends in health care delivery that emergency physicians should know as part of the core content of emergency medicine.^5^ Yet many emergency physicians are unfamiliar with the safe handling of firearms.^6^ The ACEP Policy on Firearm Safety and Injury Prevention “condemns the current rates of injury and death from firearms in the United States.”^7^ More recently, California ACEP updated its 2013 firearm injury prevention policy to reaffirm strategies such as child-protective safety and storage and extreme risk protection orders to reduce injury and death related to firearms.^8^ Unlike the broader society, as emergency physicians we have unique, first-hand experience with firearm-associated injuries and deaths. Our specialty is harmed by firearms-related violence, whether in the trauma bay or when it claims the lives of fellow emergency physicians like Drs. Tamara O’Neal and Kevin Rodgers.^9^,^10^ As emergency physicians, we can, and we must, be the example of civil, respectful, and evidence-based approaches to finding solutions to the most challenging public health problems. There is room for disagreement about firearms; more importantly, there is opportunity and responsibility for us to use our professional experiences, expertise, and perspectives to lead objective, respectful, civil, and evidence-based discussions about how to reduce disability and death from all causes, including firearms. These discussions, while uncomfortable, are squarely “in our lane.” If not us, then whom?

In full disclosure, as editors of this special issue, we are disparate with regard to firearms. One owns firearms, one does not. One lives where gun ownership is uncommon, one where ownership is common. In the context of this diversity, we share unity of purpose, and invite our emergency physician colleagues, public health and other researchers, and the broader public, to engage in civil discourse and research.

## References

[1] Kellerman AL, Rivara FP (2013). Silencing the science on gun research. JAMA.

[2] Fessenden M (2015). Why so few scientists are studying the causes of gun violence. Smithsonian Magazine.

[3] Stark DE, Shah NH (2017). Funding and publication of research on gun violence and other leading causes of death. JAMA.

[4] Greene J (2019). A balancing act: the emergency physician role in firearms safety. Ann Emerg Med.

[5] American Board of Emergency Medicine 2019 Model of the Clinical Practice of Emergency Medicine. “20.4.7.4 – Firearm injury prevention.”.

[6] Ketterer AR, Ray K, Grossestreurer A (2019). Emergency physicians’ familiarity with safe handling of firearms. West J Emerg Med.

[7] American College of Emergency Physicians (ACEP) Firearm Safety and Injury Prevention Policy Statement.

[8] Fernandez J, Nichols T, Basrai Z (2021). California ACEP firearm injury prevention policy. West J Emerg Med.

[9] Brice-Saddler M (2018). The devasting loss of the doctor killed at a Chicago hospital by her former fiancé. The Washington Post.

[10] Eales T (2018). In memoriam: Kevin G. Rodgers, MD. EM Resident.

